# Licence to Practise and Liberty to Teach Medicine in the English Provinces
*A Paper read at a Meeting of the Manchester Medical Society on Wednesday, November 7th, 1934.


**Published:** 1935

**Authors:** J. A. Nixon

**Affiliations:** Professor of Medicine in the University of Bristol; Physician to the Bristol Royal Infirmary; Consulting Physician to Southmead Hospital


### LICENCE TO PRACTISE AND LIBERTY TO TEACH MEDICINE IN THE ENGLISH PROVINCES.*

BY

J. A. Nixon, C.M.G., M.D., F.R.C.P.,

*Professor of Medicine in the University of Bristol ;*
*Physician to the Bristol Royal Infirmary ;*
*Consulting Physician to Southmead Hospital.*

---

In electing me an honorary member of the Manchester Medical Society and inviting me to address you on the occasion of the Centenary you have paid me a very great compliment for which I give you my warmest thanks. I believe there is a three-fold reason for my being thus honoured. The first and strongest is the personal friendship between your President and myself, the second was a desire to acknowledge a debt which Manchester owes to Bristol—Dr. Thomas Turner, the founder of your Medical School, was apprenticed to Nehemiah Duck, the third to emphasize the independence and interdependence of the provincial medical schools of England.

Your President asked me to address you on some subject which might bring out the importance of the provincial towns of England in the evolution of medical practice and teaching.

There is a danger lest the newness of our provincial universities should cause us to forget the important

* A Paper read at a Meeting of the Manchester Medical Society on Wednesday, November 7th, 1934.

part played by provincial towns in furnishing the public with competent and educated medical practitioners.

The fame of the capital cities of London, Edinburgh and Dublin has in recent years given rise to the belief that until the newer universities were founded the training of medical practitioners for the country at large was carried out in these capitals. This was not the case. Facilities for training medical practitioners in the capitals were never adequate to the needs of the country.

The origin of the modern system of medical education and licensing cannot be traced beyond the Benedictine School of Monte Cassino, where a monastery was founded by St. Benedict himself in 529. This was a monastic school of medicine with a hospital or infirmary for the sick. In 800 there was a school of medicine at Salerno on the model of those at Edessa or Baghdad where the Arabian physicians studied and taught. The history of medical education and regulation of medical practice in England begins in the twelfth century. This was the period immediately following the Norman Conquest, when the Universities of Oxford and Cambridge began to flourish (no matter what dates are claimed for their earliest foundations), when the craft guilds took root and the monastic Hospital of St. Bartholomew was founded in London.

Physicians were educated at one or other of the many universities which sprang up in the twelfth and thirteenth centuries just after the Crusades, and were the result of the introduction of the knowledge of the East to Western Europe.

Masters of Surgery, or Surgeons of the Long Robe as they were called in France, were similarly university graduates, men like Henri de Mondeville, Guy de Chauliac and John of Arderne, who Hæser suggests was educated at Montpellier. The Master-Surgeons formed a small guild in London as early as 1369, united for a short time with the physicians in 1423, and finally became merged into the United Company of Barbers and Surgeons in 1540. Originally they were consulting and operating surgeons, such as exist to-day. The Surgeons of the Short Robe were barbers practising surgery.

But the innumerable rabble of pretenders in medical practice led the physicians to petition King Henry V, begging amongst other requests his "hey prudence to send warrant to all the Sherrefs of England, to every practysor in Fysyk, nought graduated in the same science, that will practise forth, be withynne one of the Universities of this lond by a certayne day, that they ben able and approved after trewe and streyte examinacion be receyved to theyr degree and they that be nought able to cese from the practyse unto the tyme that they be able or never more entre mette thereof." The result of the petition is mentioned in Fuller's *History of the University of Cambridge:* "The same year (1419, Henry V) it was ordered in parliament that none should practise physic or surgery except approved on by one of the universities."

In London soon afterwards, at some date between May, 1421, and May, 1423, the physicians and surgeons formed a conjoint college to regulate the medical and surgical professions ; but after 1424 there is no further evidence of its existence, and the two branches of the profession went their several ways. The surgeons, incorporated as a Fellowship in London in 1435, taught apprentices and examined outsiders who were anxious to practise surgery.

Hitherto it will be seen that the efforts at regulating the profession had been confined to examining practitioners or aspirants to practise and issuing licences. The theory of physic was studied as a branch of polite learning, the practice of medicine and surgery was picked up from association with existing practitioners, and more especially by joining the army in France. The Fellowship of Surgeons of 1435 introduced the systematic teaching of apprentices: treatises on practical surgery had come into vogue, such as those of de Mondeville and de Chauliac, which were being translated into English. These books were handbooks for general practice. Norman Moore says: "Yet so penetrating is the influence of that great teacher, the patient, that even in the twelfth century the observing habit of the medical mind might probably have been detected."

From the time of Henry V to that of Henry VIII the history of medical education consists chiefly of a record of struggles between various authorities for the right to license medical practitioners. In London the Company of Barber-Surgeons, and later the College of Surgeons, shared with the College of Physicians the right to license practitioners in the City of London and within seven miles of the same. Yet they had grudgingly to admit another licensing authority—"except he be first examined, approved and admitted by the Bishop of London or the Dean of St. Paul's." This was the common manner of licensing in the fifteenth and sixteenth centuries. The guilds or companies shared with the bishop of the diocese the licensing authority. The bishop or his representative had to associate himself with four doctors of physic before granting a licence in medicine, and for surgery other expert persons in that faculty who were to certify after due examination as to the fitness of the candidate to practise.

Yet even the Acts of Henry VIII giving the bishops and barber-surgeons licensing rights were overridden in a few years by the Act of 1542, which laid down that it shall be lawful to every person being the King's subject having knowledge of the nature of herbs, roots and waters . . . within any part of the Realm of England or within any other of the King's dominions to practise, use and minister in and to any outward sore . . . or drinks for the stone, strangury and agues without being sued under the Act 3 Henry VIII, c. 11, for the regulation of physicians and surgeons. The reason for these exemptions is explained in the Act as being the avarice and ignorance of the most part of the Members of the Craft of Surgeons. Although it is stated that the herbalists and water-curers had hitherto not taken anything for their pains and skill, but "have ministered the same to the poor people only for neighbourhood and God's sake and of pity and charity," there is nothing in the Act to forbid them to take rewards and fees in future.

Dr. A. A. Mumford has just written me a very kind letter which throws a new light on the episcopal licences. I did not previously know that subscription to the Thirty-nine Articles was demanded of candidates for this form of licence. He describes an instance in his own family of the difficulties and the methods adopted to overcome them: "James Langley, of Isham, Northampton, born about 1699, settled at Oundle as a surgeon in 1738. He was, I judge from the books I possess with his name inscribed, a well-educated man, trained in one of the Nonconformist academies, when Nonconformists were not allowed to study at the English universities. He practised as a surgeon at Oundle until

1741, but without a bishop's licence, for he would not sign the Thirty-nine Articles, a necessary condition of obtaining their licence in the provinces. He was cited, with several others, to appear before the bishop at Peterborough in 1739. The others were licensed, but he was not licensed, being a Nonconformist. He had, however, to clear out of Oundle and went to London, but not being a member of the College of Surgeons, could only practise by living outside the city boundaries. One side of Old Street was in the boundaries, the other, 'north side,' was outside. So John Langley apparently lived on the north side of the street. His son-in-law, John Ryland, was a dealer in oils and hops, and as such was occasionally entrusted with the care of certain cases of mental derangement, treatment by oils being then rather popular. I have cited this case of my ancestors to show that it was not entirely lack of educational opportunities that delayed the progress of medical education, since medicine was one of the subjects for which students at the private Nonconformist academies were prepared, but rather the vested interests of the episcopacy, which, however, were not very frequently called into action, if you may judge by the episcopal register I have examined."

Anatomical instruction was first recognized in the Seal of Cause granted by the Town Council of Edinburgh to the surgeons and barbers in the year 1505, and confirmed by King James IV in the following year. This preceded even the law of Henry VIII in 1540, by which four bodies of executed criminals were granted to the surgeons and barbers of London. Wherever a Guild or Company of Barber-Surgeons existed similar rules for the teaching of apprentices were prescribed. Manchester, I understand, had no such guilds in Tudor and Stuart times. I am at a loss to know what facilities, if any, were offered to surgeons' apprentices in Manchester except that they could learn from their masters, and the bishop of the diocese could license them if they were found competent. Comrie states that about 1657 in Scotland anyone who desired to practise medicine or surgery might do so without let or hindrance so long as he did not invade the district in and around Glasgow supervised by the Faculty of Physicians and Surgeons or practise as a Barber-Surgeon in one of the burghs where a guild existed. The necessary qualification elsewhere consisted simply in the ability to obtain patients. The practice in Edinburgh as described by Comrie at the beginning of the eighteenth century is probably a fair representation of what was going on in England also.

"From 1657 onwards a change had taken place in the nature of the surgeon's craft. Pharmacy was now taught along with the art of surgery. Pharmacy had a greater attraction to apprentices than the barber craft, and they began to set up as surgeon-apothecaries. During the course of the seventeenth century the surgeon-apothecary became the type of practitioner who looked after the health of the community and lost all connection with the calling of the barber. His training consisted solely in apprenticeship, generally of five years to an established practitioner, although in the case of a man who wished to attain reputation and success in practice he had usually taken occasion in his youth to hear lectures at one of the universities or in some continental medical school."

The career of your great obstetric surgeon, Charles White, seems an admirable example of this way of

"graduating." Professor Adami has thus described his medical education: "There were then (he was born in 1728) no medical schools in the provinces, for Oxford and Cambridge, though they afforded occasional anatomies and gave degrees in medicine, had no adequate course of hospital study; and as for hospital opportunities in Manchester, it was Charles White himself who launched the first hospital there. Experience was gained in the main from apprenticeship, and it would seem that Charles acted as apprentice to his father. His wider education away from home began at the age of twenty, when he went up to London. The first lectures he attended were those upon Anatomy by William Hunter." In 1762, at the age of thirty-four, he was elected to the Royal Society, and in that same year was admitted a member of the Corporation of Surgeons. In 1783 a College of Arts and Sciences was established in Manchester, and he took charge of the Anatomical Department.

### EIGHTEENTH CENTURY: REVIVAL OF HOSPITALS.

The introduction of the system of unpaid attendance of the medical staffs at our voluntary hospitals is the outcome of the teaching of apprentices. For close on two hundred years after the suppression of the monasteries there was no provision of hospitals for the sick in England except in the City of London, where the Corporation was strong enough to retain the hospitals of St. Bartholomew, St. Thomas and Bethlem. Elsewhere the hospitals ceased to exist at the same time as the monasteries to which they were attached.

For some time surgeons and apothecaries were trained by the apprenticeship of students to practitioners. When their apprenticeship was finished the students were admissible to the guild (if there was one) in the town where they wished to practise. If there was no guild they presented their credentials to the bishop of the diocese or his representative and received a licence to practise. But the guilds only enjoyed their powers from the fifteenth century to the end of the seventeenth.

It was in 1468 that the barbers practising surgery obtained a charter from Edward IV. The guilds were first given charters by Henry II, but perhaps most of the guilds in the large towns, such as Bristol and Exeter, were formed in the reign of Edward III.

During the sixteenth and early seventeenth centuries the Crown, in the absence of sufficient regular taxes, was driven to raise money by the sale of monopolies, but this caused so great an opposition that all action which could be construed as restraint of trade was held to be contrary to the public interest. Amongst other monopolies licensed medical practice was condemned and anarchy prevailed.

Soon after the beginning of the eighteenth century the Barber-Surgeons and Surgeons Companies ceased to attract candidates for admission. Their monopoly of practice was gone, and with it the value of their licences.

The period of their demise, fortunately, coincided with a new impulse to found hospitals. In 1696 the Corporation of the Poor in Bristol saw the need for hospital accommodation, and St. Peter's Hospital was founded, in part by voluntary subscriptions (*e.g.* Edward Tyson), and staffed by some volunteers (Dr. Dover being one). Then in rapid succession charity hospitals were opened.

1719 Westminster.
1725 Guy's.
1728 Dublin, Jervis St. Hospital.
1729 Edinburgh Royal Infirmary.
1734 St. George's.
1736 Winchester.
1737 Bristol Royal Infirmary.
1740 London Hospital.
1743 Northampton.
1745 Middlesex Hospital founded. Barbers separated from Surgeons in London.
1751 Newcastle.
1753 Manchester.
1800 Royal College of Surgeons, London, chartered.

I think there was some connection between the Toleration Act of 1689 and the foundation of these hospitals. The Restoration meant a revival of commerce; at the same time the thinkers of the nation turned their attention to science, and both commerce and science reacted on religion. There was a good deal of religion in the science of men like Newton. A great impulse to church-building was given by the Fire of London. It made Sir Christopher Wren into the greatest of English architects: previously he had been a man of science, eminent in astronomy, almost pre-eminent in experimental medicine as a member of the Oxford Society, which presently became the Royal Society.

The revocation of the Edict of Nantes (1685) caused exiles from France to swarm into England and all Protestant countries. They brought with them treasures of character as well as skill in new crafts and industries. Immediately upon this followed the Trial of the Seven Bishops (1688) and the revolution which brought William of Orange to the throne.

The reign of Queen Anne was marked by the rising strength of England, not only in statesmanship, in wars on sea and land, but in religious thought, in philosophy, and above all in humanitarianism. This religious and intellectual revival was responsible for the foundation of hospitals all over the country, and the spirit in which they were founded can be best exemplified in the declaration contained in the "Vellum Book" of the Bristol Royal Infirmary, which was taken almost verbatim from the records of the first meeting of the promoters of the Westminster Hospital, 1719, which begins: "Whereas many sick persons languish and die miserably for want of necessaries . . . we whose names are underwritten (in Obedience to the rules of our holy Religion) desiring as far as in us lies to find some remedy for this great misery of our Poor neighbours do subscribe the following sums of money, to be by us continued yearly during pleasure for the procuring furnishing and defraying the necessary expence of An Infirmary at Bristol for the benefit of the poor sick." Here we see pure, disinterested philanthropy was the motive force in the foundation of our voluntary hospitals. Among the promoters there were always to be found medical men ready to give their professional services gratuitously to the charity. From the earliest days, however, surgeons were allowed to bring their apprentices to assist them and to receive money for teaching their pupils. In Bristol this was no inconsiderable item. One surgeon at the Infirmary received in pupils' fees £708 10s. 4d. in thirteen years from 1744 to 1757. This right to teach pupils at the hospitals which they had helped to found gave the surgeons a distinct advantage over the private practitioners unconnected with any hospital. The philanthropy which actuated the founding surgeons was supported in later times by substantial advantages both in the right to have pupils and in the prestige attaching to a position on a hospital staff. Henceforth the apprentice was rarely satisfied with seeing the work done by one master alone and that work confined to private practice. Without any force of legislation all medical students who were able arranged to "walk the hospitals" either in the capitals or in the provinces.

From the novelists' accounts we might conclude that medical students in the eighteenth and early nineteenth centuries were illiterate young men of low social standing. Munro Smith has taken pains to show that this was not the case in Bristol, and probably not in other centres: "The authorities of the Bristol Infirmary were extremely particular as to the class of youth taken, and if their general education was not considered sufficiently good they were refused. Thus in 1766 the son of a Mrs. Ford was not admitted because his schoolmaster could not give a sufficiently good report of his studies, and he was sent back to school for another year. They were frequently the sons of gentlemen of good position; in fact, amongst the pupils and officials at the Bristol Infirmary, especially during the first hundred years of its existence, the standard as to social position was very high."

I must apologise for saying so much about Bristol, but our records on the subjects are unrivalled. We possess at the Royal Infirmary fourteen bulky manuscript volumes containing written accounts of Infirmary affairs—elections, lists of officers, notices of meetings, newspaper cuttings and historical memoranda, together with biographical histories of many of those who were connected with the institution from the time of its inception (1736) to the year 1842. These books were collected by Richard Smith, who was Surgeon to the Infirmary from 1796 to 1843. There are hundreds of biographical histories, and amongst them are many records of the pupils and apprentices.

Whilst the opportunities for study were vastly improved by the increasing number of towns where teaching was systematically undertaken, scarcely any attempt was made by the Government to see that medical practitioners were qualified to practise. The guilds had lost their authority, the bishop's licence was no longer required, and the areas of jurisdiction of the colleges of surgeons in the capitals were very restricted. Doctors for the army in Marlborough's wars and the navy were examined as far as could be insisted by the college and the halls, but readers of *Roderick Random* may gather that in some instances the examination was not very searching.

I believe the Slave Trade called for closer supervision, and the professional standing of many of the "African Surgeons" was exceedingly high, notably Falconbridge, whose evidence before the Parliamentary Committee was one of the most powerful factors in bringing about the Abolition of the Slave Trade. In this connection we find an Act passed in 1789 requiring surgeons on the "African" ships to produce certificates of having passed an examination at Surgeons Hall in London or at the College of Surgeons of Edinburgh or Dublin, or at "some publick or county hospital." Under this Act the surgeons of the Liverpool Infirmary met once a month regularly between 1789 and 1807, examining 634 candidates and passing rather less than 500. I cannot discover records of any other public or county hospital exercising this right, though I suspect that Falconbridge, a student of the Bristol Infirmary, became an "African

Surgeon " on some Bristol recommendation without any diploma.

There seems to have been very little attempt made to license private medical practitioners. The qualification of a doctor was ability to get patients ; yet there were obvious advantages in having studied in recognized hospitals under competent teachers, and very shortly after the revival of hospitals the hospital surgeons began to form private medical schools where anatomy was taught regularly. There was keen competition to form these schools in the great provincial cities such as Manchester, where schools of medicine or anatomy were started.

In 1800 the Royal College of Surgeons in London was granted its charter, and aimed at obtaining a monopoly for teaching and licensing all surgeons in the realm. It was given practically no compulsory powers, and unlicensed practice flourished unchecked. The licence of the college was optional, but the numerous independent schools quarrelled for recognition and the right to prepare candidates for examination by the college.

In addition to the practitioners licensed by the Royal College of Surgeons there were many general practitioners apprenticed under the Society of Apothecaries. The Society of Apothecaries of London was incorporated in 1606, in union with the Grocers Company. A new and independent charter was granted in 1617. Modern medical education in England we owe to the Apothecaries Society. It was the first body to appoint examiners in medicine ; but more than this, it recognized medical and surgical teaching in the provinces, including clinical work in provincial hospitals. This attitude the Society maintained in spite of the most obstinate opposition from the Royal College of Surgeons. How important this action by the apothecaries was is demonstrated in Professor Hay's Liverpool centenary address: "In 1821 the Society of Apothecaries recognized the lectures of Dr. Joseph Jordan of Manchester as fulfilling their requirements, and three years later, in 1824, the clinical teaching of the Manchester Infirmary School was accepted as meeting London regulations. Manchester has the honour of being the first provincial medical school in England. The two men responsible were Dr. Jordan and Dr. Thomas Turner, both of Manchester." Thomas Turner had been apprenticed to the Bristol surgeon-apothecary Nehemiah Duck.

The apothecaries could take no fees for attendance, but might charge for their medicines. Although many of them were skilful practitioners, there were more whose sole object was to prescribe (and charge for) as many bottles of medicine as their patients would accept. Even in modern times we know that no consultation is considered quite satisfactory without a bottle of medicine.

There is an interesting sidelight thrown on their general efficiency by the remarks made at a conference reported in the *Lancet* in 1844: "Mr. Astley, one of the oldest licentiates of the Company of Apothecaries, could well remember the condition of the general practitioner previous to 1815. So ignorant were they in the country that he recollected an instance of a man of fortune and influence having to send thirty miles for a surgeon to pass a catheter for him."

At length the apothecaries in 1815 obtained an Act of Parliament enabling them to hold an examination for all England and to prosecute unqualified apothecaries. The provincial medical schools then began to claim recognition to prepare candidates for the apothecaries' examination and licence. The College of Surgeons refused to recognize the provincial hospitals as complete clinical schools, and insisted that all candidates for its examination should spend some time "walking" a London hospital. The idea of the college was to make all candidates come to London, and its opposition to the claims of the provincial medical schools was unrelenting.

But the London surgeons reckoned without the apothecaries and the powers conferred on them by the Act of 1815. In 1821 Jordan's lectures in Manchester were recognized, and the barrier against provincial teaching was broken down. In Bristol one of the schools was recognized in 1828, and it became a complete school in 1833: all the work required by the Apothecaries' Society could be carried out in Bristol, whilst the College of Surgeons demanded six months' work in London. The college, faced with the prospect of provincial candidates ignoring its licence and preferring that of the apothecaries, was driven to surrender to Manchester in 1834, and after that date the provincial medical schools' right to carry through the whole curriculum was unquestioned. In 1836 registration of births and deaths was made compulsory by Act of Parliament, and in 1858 The Medical Act was passed, which, whilst it did not forbid unqualified practice, allows no practitioner to recover fees, hold public appointments, or sign certificates unless he has been properly examined and placed on the Register. Henceforward, as death registration had been made compulsory, competence to sign death certificates became necessary for practice.

The open-mindedness of the Apothecaries' Society had established once and for all the provincial medical schools, but its influence was going to be once more exerted in an unexpected way. When the Register set up by the 1858 Act was opened a woman doctor, Miss Elizabeth Blackwell, who was born in Bristol, made successful application to be entered on the English Register by virtue of her American medical degree (M.D. Geneva). This did not open the door for other women practitioners unless they could secure a medical education and a licence from some British authority. Miss Elizabeth Garrett (Anderson), having applied without success to the other examining and licensing bodies, was in 1862 admitted to the examinations of the Apothecaries' Society, and eventually obtained its licence to practise, which entitled her to be placed on the English Register. The Society of Apothecaries in those early days of the nineteenth century had no rival for enlightenment and broad-mindedness. The provincial schools owe their whole existence to that Society, whilst some of us even hold it in high esteem for throwing open the medical profession to women.

In 1880 the Victoria University was the first in the provinces to receive its charter. By a natural corollary the right to confer medical degrees was shortly (in 1883) granted to that university in Manchester. The year 1880 saw also the abolition of medical apprenticeship.

By slow degrees the responsibility for teaching and the right to license have tended to be controlled by the same educational authority. All modern provincial universities in England owe their medical faculties to medical schools which were founded by the generosity and personal ability of individual members of unpaid hospital staffs. No university outside of London and

Edinburgh has ever founded a hospital or taken any share in the institution of a medical school. But wherever a medical school has survived to become an integral part of a university it will be observed that a medical society, such as the Manchester Medical Society, was founded about the same time and played an important part in fostering the growth of the young medical school. Some of the societies have not survived, but you are to be congratulated that the Centenary of your Society is almost contemporary with the Centenary of your Medical School.

My personal view is that the licensing corporations with no responsibility for teaching have come to the end of their useful existence. They do not now contribute in any way to the progress of medical education. But there is an increasing danger that they may multiply, for the sake of fees, the issue of diplomas in special subjects in which they offer no instruction. This tendency should, I believe, be very closely watched and discouraged. If specialist diplomas are necessary it is essential that they should only be issued by corporations which accept responsibility for giving instruction.

To illustrate my meaning, let me point out to you that already two classes of specialist diplomas exist. Some of these diplomas are issued by universities or schools which can offer facilities for study in particular branches of medicine. Such courses of study may well be encouraged. There are, however, others issued by what must be designated as educationally irresponsible bodies. The time has come for irrevocably combining the licence to practise with the liberty to teach, and I hold that the right to grant licences to practise should be withdrawn from any corporation or authority which is not actively responsible for affording educational facilities. The privilege of educating recruits to our profession is more important than the responsibility for examining and licensing.* I believe that the standard of medical education in our provincial schools is in no way inferior to that in our capitals. I make no apology for holding these views, which I admit take their origin from a former Master of the College in Cambridge to which I have the honour to belong. Dr. Dell, who was intruded at the Commonwealth as Master of Gonville and Caius College, was the first to suggest that there might be other universities than those of Oxford and Cambridge, and I will conclude with a few quotations from one of his sermons :—

"I conceive it meet, that the civil power, or chief magistrates, should take great care of the education of youth . . . inasmuch as what the youth now is, the whole commonwealth will shortly be." He desires schools throughout the whole nation "not only in cities and great towns, but also, as much as may be, in all lesser villages." "It may be convenient also, that there may be some universities or colleges, for the instructing youth in the knowledge of the liberal Arts . . . also . . . the studies of physic, and of the

* Opinions have differed violently on this question, as indicated by a letter in the *Lancet*, vol. 34 (1839), p. 384, from Dr. George F. Hayden, who wrote :—

"We medical reformers in Ireland have long felt the grievous effects of a union of licensing and teaching in one and the same body." He quotes a petition to the late King (William IV) sent by the London College of Surgeons when London University was seeking for a Charter to enable them to examine candidates and grant degrees in medicine :

"Your petitioners are firmly convinced that the occupation of teaching and the power of examining and conferring degrees ought to be exercised as they now are by distinct institutions, and that the union in the one and the same institution of those discordant attributes must be attended with danger to the public welfare." *Discordant attributes* is a happy phrase !

### LICENCE TO PRACTISE AND LIBERTY TO TEACH.

| Licensing. | | Teaching. |
|---|---|---|
| A.D. | St. Benedict, 480–543. | A.D. 529. Monastic medical teaching including hospital practice, instituted at Benedictine Monastery of Monte Cassino. |
| 1140. Salerno, licences to practise. | Charlemagne, 742–814.<br>Haroun-al-Raschid, 763–809.<br>Alcuin of York, 736–804. | 800. Arabian School of Medicine at Salerno. |
| 12th Century.—Rise of Craft Guilds. | Henry I, 1100, to John, 1199. | 12th Century.—Barber-Surgeons instructed their apprentices.<br>Pupillage to established practitioners was customary from this time to 1880. |
| | 1209. 3,000 students and Professors withdrew from Oxford to Cambridge.<br>"So that not one remained of all the University."—*Fuller*. | Universities taught medicine without hospital practice until Edinburgh University founded the Infirmary in 1729. |
| | | 1123. Monastic medical teaching with hospital practice established in London.<br>St. Bartholomew's founded. |
| 1363. Act compelling all surgeons to belong to one of the Guilds. | Edward III, 1327–1377.<br>The Black Death, 1348. | 1315. Mondini's first demonstration of Anatomy at Bologna. |
| 1421. First law regulating medical practice.<br>"No one to practise in physic unless approved by the Universities and in surgery by the Masters in that Art." | Henry V, 1413–1422.<br>Perpetual Peace of Troyes, 1420. | |
| 1423. Conjoint Society of Military Surgeons and Physicians founded but short lived. | | |
| | James IV of Scotland, 1488–1513.<br>Henry VII, 1485–1509. | 1506. Anatomical instruction made compulsory for surgeons and barbers in Edinburgh.<br>(Seal of Cause to Guild of Surgeons and Barbers at Edinburgh by James IV.) |
| 1511. Bishops' licences instituted. | Henry VIII, 1509–1547. | 1536. Suppression of Monasteries. |
| 1524. Licence granted by T. Cromwell to Roger Smyth, citizen and grocer of London, to practise physic and surgery in any part of the realm. | | |
| 1542. Herbalists permitted to practise in any part | | |

| | | |
|---|---|---|
| *1617. Apothecaries Society, London, granted separate Charter.* | *James VI of Scotland and I of England. 1603–1625* | *16th and 17th Centuries.—Educational system of Barber-Surgeons flourished. Apprenticeship to established practitioners supplemented by Guild lectures and demonstrations of Anatomy in towns where Guilds existed, but without hospital practice.* |
| 1667. Licences granted to Mountebanks to vend medicines and practise medicine and surgery in any city, town or borough in the Kingdom. | Charles II, 1660–1685.<br>Royal Society founded, 1662.<br>Revocation of the Edict of Nantes, 1685.<br>James II, 1685–1688.<br>Revolution, 1688.<br>Act of Toleration, 1689. | |
| | | 1696. Revival of Hospitals.<br>St. Peter's Hospital, Bristol (Poor Law).<br>1719. Westminster Hospital.<br>1737. Bristol Infirmary.<br>1753. Manchester Infirmary.<br>(1719–1753: Charity Hospitals.) |
| 1789. Act requiring Ship Surgeons to produce certificate of having passed examination at Surgeons Hall in London or at the College of Surgeons of Edinburgh or Dublin or at *some public or county hospital.* | George III, 1760–1820. | 18th Century.—Apprenticeship to hospital-surgeon or apothecary.<br>Practitioners chose to be apothecaries rather than surgeons (or barber-surgeons).<br>Physicians encouraged apothecaries and introduced them to their patients. |
| 1815 Apothecaries Act. | George IV, 1820–1830. | 1821. Apothecaries Society recognized provincial medical schools: *e.g.*, Jordan's.<br>1824. Manchester, the first complete provincial medical school.<br>1834. Clinical teaching at Manchester Infirmary recognized by the College of Surgeons.<br>Manchester Medical Society founded. |
| 1858. Medical Registration Act.<br>1859. Elizabeth Blackwell admitted to English Register (on American degree).<br>1862. Elizabeth Garrett (Anderson) admitted to the examination of Apothecaries Society. | Victoria, 1837–1901. | 1880. Apprenticeship abolished. Victoria University founded.<br>1883. First provincial medical degrees (Manchester). |

law, according to the reformation which a wise and godly authority will cause them to pass under, both being now exceedingly corrupt and out of order, both for practice and fees." "But why these Universities or Colleges should be only at Cambridge and Oxford, I know no reason. . . . Doubtless it would be more suitable and more advantageous to the good of all the people, to have Universities or Colleges, one at least, in every great town or city in the nation, as in London, York, Bristol, Exeter, Norwich and the like. . . ." He argues "there will then be no such need of endowment of scholarships; inasmuch as the people having colleges in their own cities, near their own houses, may maintain their children at home, whilst they learn in the schools; which would be indeed the greatest advantage to learning that can be thought of."

